# Pseudomembranous Colitis: Not Always Caused by *Clostridium difficile*

**DOI:** 10.1155/2014/812704

**Published:** 2014-08-18

**Authors:** Derek M. Tang, Nathalie H. Urrunaga, Hannah De Groot, Erik C. von Rosenvinge, Guofeng Xie, Leyla J. Ghazi

**Affiliations:** Division of Gastroenterology & Hepatology, Department of Medicine, University of Maryland School of Medicine, Baltimore, MD 21201, USA

## Abstract

Although classically pseudomembranous colitis is caused by *Clostridium difficile*, it can result from several etiologies. Certain medications, chemical injury, collagenous colitis, inflammatory bowel disease, ischemia, and other infectious pathogens can reportedly cause mucosal injury and subsequent pseudomembrane formation. We present the case of a middle-aged woman with vascular disease who was incorrectly diagnosed with refractory *C. difficile* infection due to the presence of pseudomembranes. Further imaging, endoscopy, and careful histopathology review revealed chronic ischemia as the cause of her pseudomembranous colitis and diarrhea. This case highlights the need for gastroenterologists to consider non-*C. difficile* etiologies when diagnosing pseudomembranous colitis.

## 1. Introduction

Pseudomembranous colitis is commonly associated with* Clostridium difficile* infection (CDI) but can be a consequence of other disease processes. Mucosal necrosis leads to pseudomembrane formation in both CDI and ischemia, but the two entities can be distinguished by endoscopic and histologic appearances of the colon [1]. Occlusive arterial and venous thromboemboli can cause ischemic colitis (IC), but hypoperfusion without occlusion of the mesenteric or the internal iliac arteries is the main mechanism. Low blood flow states, such as atherosclerosis and septic shock, affect the “watershed” areas, which comprise the splenic flexure and rectosigmoid junction. Patients with IC have varied presentations that depend on the onset and duration of injury and extent of involvement. Although patient risk factors, imaging, and clinical presentation can raise suspicion for colon ischemia, arteriography and colonoscopy with biopsies remain the mainstays of diagnosis.

## 2. Case Report

A 65-year-old woman presented with a 3 months of diarrhea. Her past medical history was significant for peripheral vascular disease (PVD), diabetes, myocardial infarction with percutaneous intervention, and ischemic cardiomyopathy. She did not have any abdominal discomfort, blood in the stool, fever, lactic acidosis, or leukocytosis. Physical exam revealed a soft nontender and nondistended abdomen with normal bowel sounds.

Initial laboratory evaluation of diarrhea showed too numerous to count fecal leukocytes and negative stool culture. Tests for infectious pathogens (*Campylobacter*,* Cryptosporidium*,* Cyclospora*,* Giardia*,* Isospora*,* Escherichia coli* 0157:H7,* Salmonella*, and* Shigella*) were negative. Enzyme immunoassay for toxins A and B and polymerase chain reaction testing, for CDI, were repeatedly negative. Serum levels for calcitonin, chromogranin A, gastrin, serotonin, somatostatin, thyroid stimulating hormone, and vasoactive intestine peptide were within normal limits. Urinary concentration of 5-hydroxyindoleacetic acid was unremarkable. Antibody tests for celiac disease were negative. Erythrocyte sedimentation rate and C-reactive protein were not elevated.

**Figure 1:**
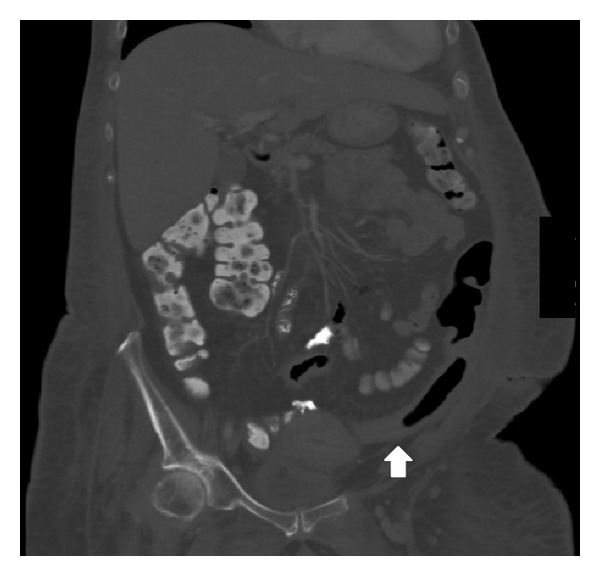
CT of the abdomen/pelvis showing mild wall thickening and an ahaustral appearance of the distal colon with pericolonic fat stranding consistent with colitis.

Computed tomography (CT) of the abdomen and pelvis showed mild wall thickening of the distal colon with infiltration and fat stranding (Figure 1). A flexible sigmoidoscopy was performed and revealed scattered and raised off-white plaques with patches of normal- appearing mucosa in the rectosigmoid colon. The pathology revealed fibrinoid material with necrotic epithelial cells, fibrin, mucus, and neutrophils consistent with pseudomembranes. The patient was started on intravenous metronidazole for empiric treatment of CDI. Her diarrhea persisted after one week of metronidazole, and oral vancomycin was initiated. The patient's diarrheal symptoms were unchanged three weeks later, and she was transferred to our tertiary medical center for consideration of fecal transplantation for treatment of refractory CDI.

**Figure 2:**
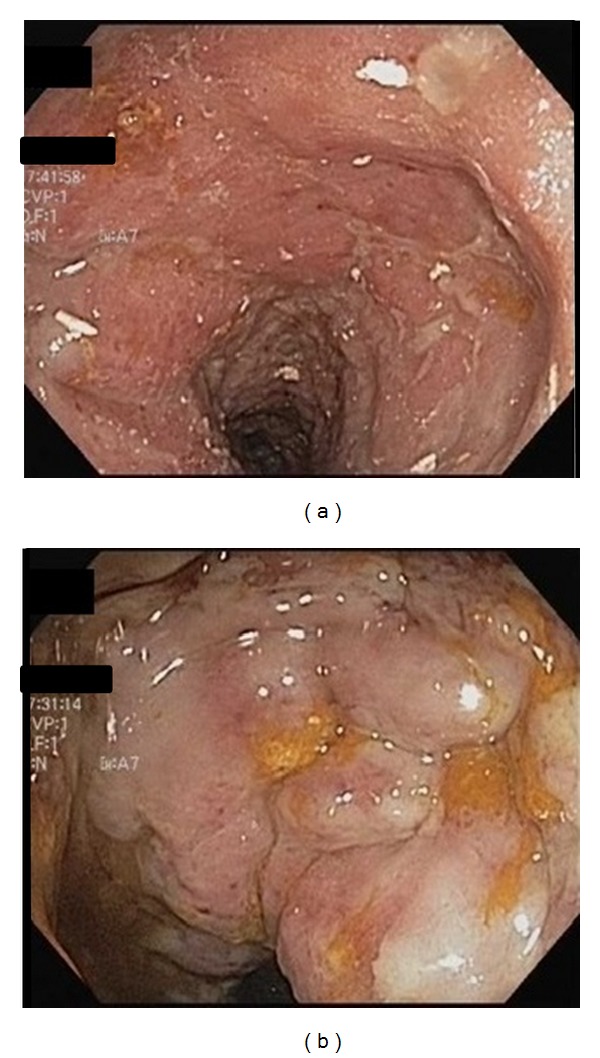
Images from repeat flexible sigmoidoscopy showing (a) erythematous, friable, and ulcerated mucosa in the rectum and (b) edematous and ulcerated mucosa with decreased vascular pattern in the sigmoid colon.

**Figure 3:**
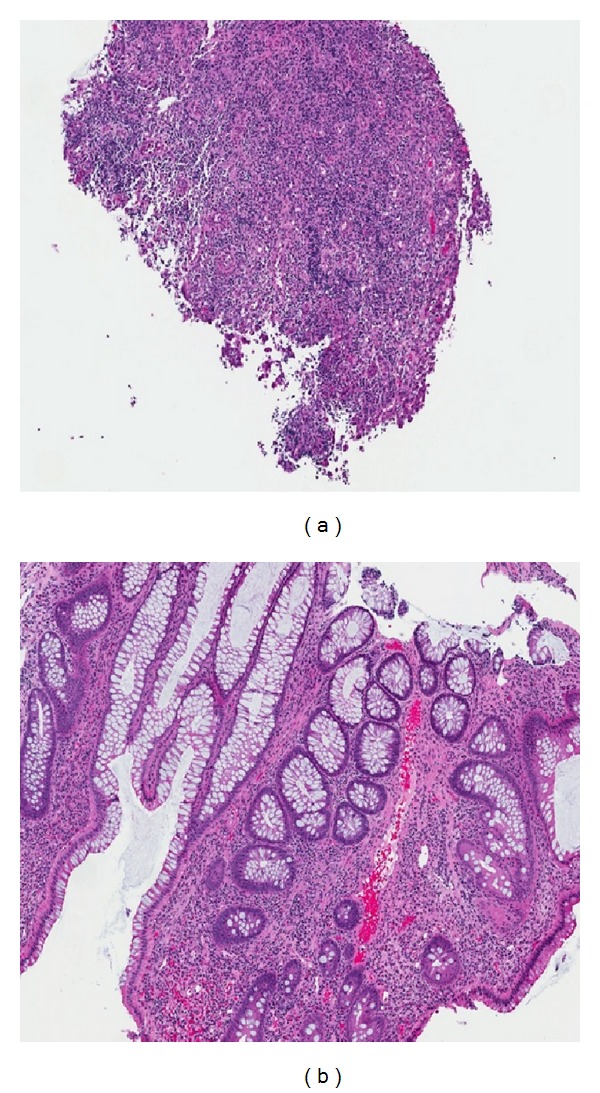
Photomicrographs of rectosigmoid mucosal biopsies demonstrating (a) an ulcer bed with loss of epithelium and extensive granulation tissue and (b) chronic ischemic colitis with architectural distortion, crypt atrophy, and hyalinization of the lamina propria (H&E stain, 20x).

Complications of the patient's PVD postponed additional gastrointestinal evaluation. She required multiple surgical debridement procedures for necrotic skin ulcers on her lower extremities. Anticoagulation and thrombolytic therapy were also given for treatment of a left popliteal artery thrombosis. Gastroenterology consultants recommended mesenteric duplex imaging, which revealed a 60–99% stenosis of the inferior mesenteric artery and a patent superior mesenteric artery. As a result of ongoing large volume diarrhea and fecal incontinence, a repeat flexible sigmoidoscopy was completed and showed severely friable, edematous, and ulcerated mucosa involving the sigmoid colon, rectosigmoid colon, and proximal rectum (Figure 2). Previously seen pseudomembranes were not visualized. Histology was characterized primarily by crypt atrophy and lamina propria hyalinization, which supports a diagnosis of chronic ischemic colitis (Figure 3). The diarrhea significantly improved with addition of loperamide. Vascular intervention was not recommended due to poor operative candidacy, and the patient is currently being evaluated for a partial colectomy.

## 3. Discussion

Pseudomembranous colitis is typically associated with CDI colitis, but it has been attributed to other inflammatory and noninflammatory states. In the literature, collagenous colitis, glutaraldehyde exposure, infectious organisms (*Campylobacter*, cytomegalovirus,* Escherichia coli* 0157:H7,* Salmonella*, and* Strongyloides*), inflammatory bowel disease, ischemia, and medications (nonsteroidal anti-inflammatory drugs, vasopressin) have been implicated as potential causes [1–7]. Through similar mechanisms of endothelial damage with impaired blood flow and oxygenation, these conditions can predispose to pseudomembrane formation and can appear endoscopically and histologically similar [8].

Colon ischemia is the most common form of intestinal ischemia and usually affects the elderly or debilitated patients with multiple comorbidities [9]. IC can present as a broad spectrum of injury, from reversible submucosal or intramural colitis to irreversible chronic ulcerating colitis with stricture or gangrene [10]. A delayed diagnosis can lead to life-threatening consequences, and thus, timely diagnosis and treatment are imperative. Diagnosis of IC is based upon history, physical examination, risk factors (e.g., aortoiliac surgery, diabetes, and heart disease), imaging, and endoscopic and pathologic evidence [11].

The mucosa and submucosa of the colon are most susceptible to hypoxia due to high metabolic demands [12]. On endoscopic examination, mild ischemia is characterized by granular mucosa with decreased vascularity. In severe cases, there is friable, edematous, and sometimes ulcerated or hemorrhagic mucosa. Furthermore, IC is often well-demarcated from normal mucosa, and only a segment of the colon is typically involved [8]. Punctuate pseudomembrane formation is seen in early ischemia, but as injury progresses, confluent pseudomembranes may be visualized. These pseudomembranes are composed of acute inflammatory cells and fibrin [13]. In the resolution phase, patchy ulceration is noted and may be similar in appearance to that seen in inflammatory bowel disease [8]. In our case, pseudomembranes were not identified on repeat endoscopy possibly because the short duration of anticoagulation and thrombolytic therapy our patient received provided some reperfusion.

Microscopic characteristics of colon biopsies help differentiate IC from CDI-associated colitis and other colitides. One prior study showed that the presence of a hyalinized lamina propria in pseudomembranous colitis was both a sensitive and a specific marker for IC [1]. Moreover, although not as specific, crypt atrophy was seen almost exclusively in IC [1]. Lamina propria hemorrhage, full-thickness mucosal necrosis, and layering of pseudomembranes in a limited colon distribution were also suggestive of an ischemic origin [1]. Both hyalinization of the lamina propria and atrophic crypts were seen on biopsies from the repeat flexible sigmoidoscopy in our patient. These histologic characteristics were not demonstrated on initial endoscopic biopsies from the referring hospital due to inadequate depth of mucosal sampling.

## 4. Conclusion

Our case report highlights the importance of awareness that pseudomembranous colitis is not always caused by CDI. Exclusion of ischemia and other etiologies is important in making an accurate diagnosis and initiating appropriate management.
